# Improving Recovery of Irritant Hand Dermatitis in Healthcare Workers With Workplace Interventions During the COVID-19 Pandemic

**DOI:** 10.3389/fpubh.2022.844269

**Published:** 2022-07-18

**Authors:** Alicia S. T. Loi, Zeenathnisa M. Aribou, Yuke Tien Fong

**Affiliations:** ^1^Department of Occupational and Environmental Medicine, Singapore General Hospital, Singapore, Singapore; ^2^Preventive Medicine, National University Health System, Singapore, Singapore

**Keywords:** COVID-19, healthcare workers, occupational dermatitis, workplace intervention, quality improvement

## Abstract

**Introduction:**

Occupational hand dermatitis is common among healthcare workers, with increased incidence during the COVID-19 pandemic. Irritant contact dermatitis accounts for the majority of occupational hand dermatitis and is largely due to frequent contact with hand hygiene products. Long-term prognosis of occupational contact dermatitis is often very poor. This study aims to identify and implement suitable workplace interventions to aid in the recovery of occupational irritant hand dermatitis among healthcare workers during the COVID-19 pandemic.

**Methods:**

A quality improvement (QI) project was performed in a tertiary hospital using the Plan-Do-Study-Act model. Healthcare workers seen at the Occupational Dermatology Clinic from March 2020 to May 2021 for the first time for likely occupational irritant dermatitis were targeted for the project. Four workplace interventions were implemented: (a) substitute current alcohol-based hand rub (ABHR) with a different, gentler ABHR, (b) alternate ABHR with gentle hand wash products, (c) temporary job modification with less clinical work (d) switch latex gloves to nitrile gloves. The improvement was assessed after 2 months of workplace intervention using a visual analogue scale, based on changes seen on photographs taken at the baseline and monthly review. The target improvement was set at 70% after 2 months of workplace interventions.

**Results:**

A total of 21 participants were included in the QI project. All participants were found to have significant improvement in their hand condition. The estimated mean reduction of signs and symptoms was 80% in comparison to their baseline hand condition before intervention.

**Conclusion:**

Workplace interventions such as substituting irritant hand hygiene products with gentler alternatives and temporary reduction in clinical duties may be useful in improving the recovery rate of irritant hand dermatitis among healthcare workers. Areas with high hand hygiene workload or high incidences of hand dermatitis may opt to implement systemic workplace changes.

## Introduction

Hand dermatitis is common among healthcare workers with reported prevalence ranging from 21 to 55% across different studies (1–4). Hand dermatitis in healthcare workers can be largely attributed to repeated hand hygiene activities, such as hand washing and the use of hand sanitizers which are known irritants (5).

Irritant contact dermatitis (ICD) accounts for 80% of occupational contact dermatitis (5–7). Cumulative exposure to irritants from hand washing and hand hygiene products directly damages the skin surface, initiating a cascade of inflammatory changes (5, 7, 8). Allergic contact dermatitis (ACD) contributes to the remaining 20% of occupational dermatitis (6). Preservatives, fragrances, and antimicrobial agents found in hand hygiene products, as well as latex and rubber accelerators in latex gloves may cause allergic reactions (5, 9, 10). Prolonged use of gloves was also associated with adverse reactions of the hands (11, 12).

Infection prevention measures were enhanced across multiple settings during the COVID-19 pandemic. In the healthcare setting, the pandemic has resulted in increased hand hygiene activities and prolonged use of personal protective equipment among its workers from high patient load and heightened infection prevention activities. Combined with insufficient downtime for skin recovery and inadequate moisturising of hands, healthcare workers are at higher risk of developing occupational contact dermatitis during the pandemic (5, 13, 14).

The long-term prognosis of occupational contact dermatitis is often very poor due to continuous exposure and can negatively impact the workers (15). A study reported recovery of occupational skin disease in only 28% of healthcare workers 6 months after diagnosis (16). Prolonged dermatitis not only affects the quality of life and work productivity, it can also be a barrier to hand hygiene compliance (17). Colonisation of skin surfaces with microorganisms is also more common in damaged skin, posing a potential risk for nosocomial infection transmission (18).

We aim to identify and implement suitable workplace interventions to aid in the recovery of occupational irritant contact dermatitis among healthcare workers during the COVID-19 pandemic. Although principles of workplace management such as identification and avoidance of precipitants, workplace educational programmes, and use of hand protection with gloves and barrier creams have been widely suggested, the effects of direct workplace interventions have yet to be adequately researched (19). The findings of this study can aid healthcare institutions in implementing workplace changes as part of the management of occupational ICD among their workers.

## Methods

The study was performed as a quality improvement (QI) project at a tertiary hospital in Singapore during the COVID-19 pandemic. The QI project team comprised of an Occupational Medicine (OM) physician and OM trainees. The target population of the project was healthcare workers seen at the Occupational Dermatology Clinic for likely occupational ICD. All healthcare workers seen at the clinic for their first consultation from March 2020 to May 2021 were included in the QI project as part of the occupational management for their skin condition, with their consent.

**Figure 1:**
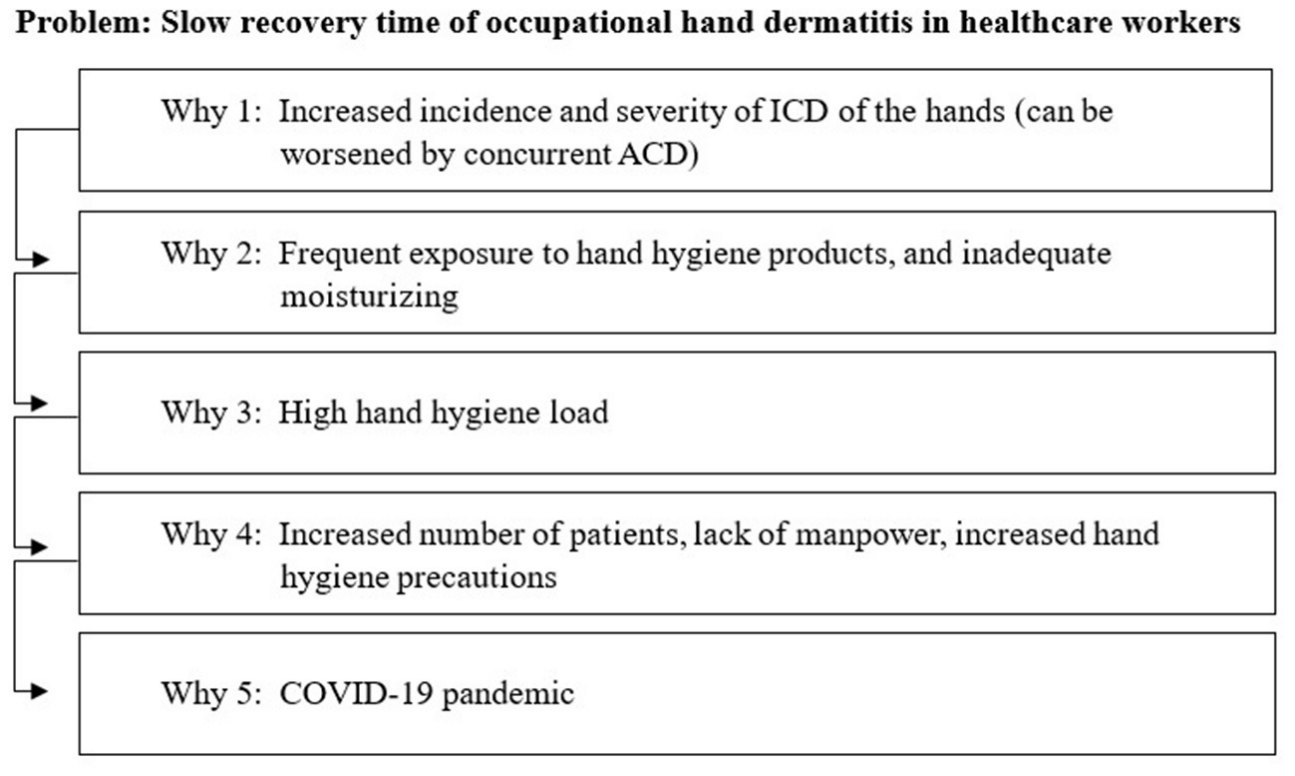
Root-cause analysis using the 5 Whys technique.

The Plan-Do-Study-Act (PDSA) model was utilised for the conception and implementation of the project (20). During the “Plan” component of the PDSA cycle, a root cause analysis based on the 5 WHYs model, identified causes of slow recovery time for ICD amongst healthcare workers (Figure 1) (21). The identified root causes were: (i) frequent exposure to hand hygiene products, (ii) inadequate moisturising, (iii) high hand hygiene load, and (iv) others (e.g., allergic contact dermatitis). Healthcare workers were also asked about the presence of the identified root causes to further quantify the frequency of these factors.

**Figure 2:**
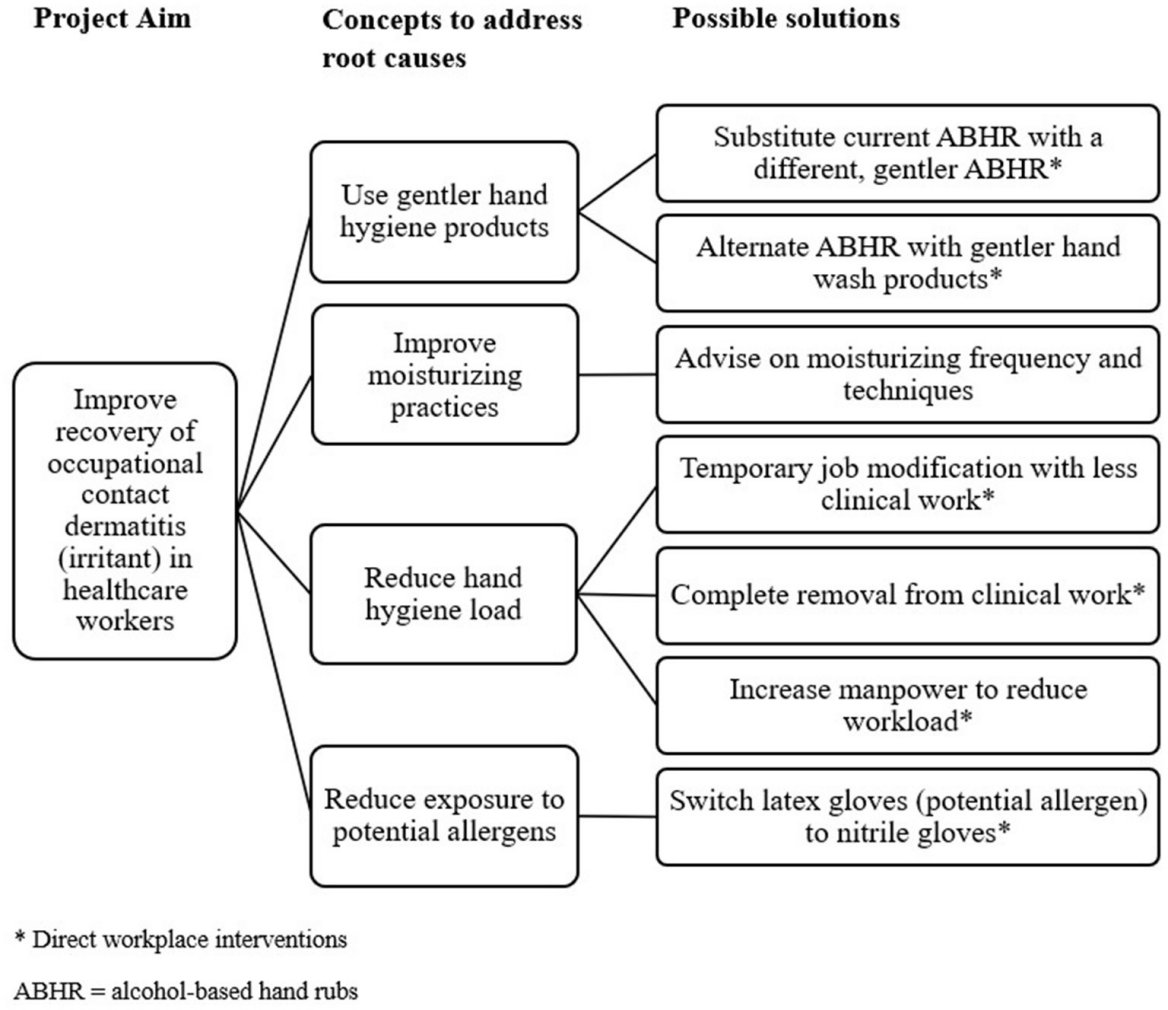
Generating solutions with a tree diagram.

During the “Do” phase of the PDSA cycle, the team proposed possible direct workplace interventions to tackle the identified root causes. These interventions were generated and scored by the team members based on four different criteria: effectiveness, feasibility, sustainability, and low cost (Figure 2, Table 1). Each criterion was scored between 1 and 5, with 1 for poorly meeting the criteria and 5 for meeting the criteria well. The effectiveness of control measures was considered based on the principles of the hierarchy of controls from the National Institute for Occupational Safety and Health (NIOSH) (22). Feasibility and sustainability were scored based on the relative ease of implementation for short-term and long-term periods. The cost of interventions was scored based on estimated expenditures or resources required to replace current products or manpower. Possible solutions scoring 15 and above were included in the programme.

**Table 1:** Prioritisation matrix for possible workplace interventions.

**Possible workplace interventions**	**Effectiveness**	**Feasibility**	**Sustainability**	**Low cost**	**Total score**
Substitute current ABHR with a different, gentler ABHR	4	5	5	5	19^*^
Alternate ABHR with gentler hand wash products	3	5	5	5	18^*^
Temporary job modification with less clinical work	4	4	3	4	15^*^
Complete removal from clinical work	5	2	1	2	10^Δ^
Increase manpower to reduce workload	4	1	2	1	8^Δ^
Switch latex gloves (potential allergen) to nitrile gloves	3	5	5	5	18^*^

*Scoring: 1—meets criteria poorly. 5—meets criteria well*.

*ABHR = alcohol-based hand rubs*.

* *Included in the programme*.

Δ *Excluded from the programme*.

Four workplace interventions were chosen for the programme:

(a) Substitute current alcohol-based hand rub (ABHR) with a different, gentler ABHR
(b) Alternate ABHR with gentle hand wash products
(c) Temporary job modification with less clinical work
(d) Switch latex gloves (potential allergen) to nitrile gloves

Workplace interventions (a), (b), and (d) were implemented for all participants while workplace intervention (c) was only implemented for participants with moderate-severity hand dermatitis, due to the reduced sustainability of the intervention. The participants were given medical letters addressed to their direct superiors for implementation of the workplace interventions.

All participants were advised on hand hygiene care, such as moisturising techniques and avoidance of household irritant products. All participants were prescribed similar topical creams including topical steroid cream and moisturisers as part of their standard care of treatment.

The hospital generally used one type of ABHR, which consists of 100% ethanol, 1-propanol, emollient, moisturiser, and fragrances. The proposed substitute ABHR consists of 70% ethanol, emollients, and moisturiser, and was readily available at the hospital. It was considered to be a gentler alternative based on lower alcohol concentration and positive response from other healthcare workers seen at the Occupational Dermatology Clinic previously, before the COVID pandemic. A mild, germicidal wash lotion with added moisturiser was proposed for the hand wash alternative.

The team subsequently implemented the proposed interventions with the support of key stakeholders such as team supervisors and patients.

The results were analysed during the “Study” phase of the PDSA cycle. Photographs of the hands were taken at the first visit and during subsequent clinic reviews at one-monthly intervals. The same OM physician assessed the degree of improvement by comparing the hand condition during clinic reviews against photographs of the hands during the first visit. The percentage improvement is based on the change from a visual analogue scale (23). Zero percent constitutes no improvement, and 100% improvement meant complete recovery. All assessments of improvement were approximate in nature, in relation to the signs and symptoms of hand dermatitis in the participants.

The target improvement for the QI project was set at 70% after 2 months of workplace interventions. Baseline comparison was deemed as no improvement (0% improvement) in ICD without workplace interventions, which was based on previous reviews of healthcare workers seen at the Occupational Dermatology Clinic with ICD.

The effects of the workplace interventions, future plans, and possible impact were discussed in the “Act” phase of the PDSA cycle.

The workplace interventions were performed as part of the standard occupational management for the participants presented to the Occupational Dermatology Clinic. The interventions were implemented systematically as a QI project to improve the recovery of hand dermatitis among healthcare workers and were approved by the hospital's Quality Improvement Committee. All the participants gave their verbal and written consent to be included in the QI project.

## Results

A total of 21 participants were included in the project, consisting of medical doctors, nurses and allied healthcare workers (Table 2). The majority of participants were female nurses, in the age range of 21–30 years old.

**Table 2:** Demographics of participants.

**Characteristics**	**Number, *n* (%)**
**Job**	
Medical doctor/medical student	2 (9%)
Nurse/nursing student	13 (62%)
Allied healthcare worker	6 (29%)
**Age (years)**	
≤ 20	5 (24%)
21–30	11 (52%)
31–39	4 (19%)
≥40	1 (5%)
**Gender**	
Male	4 (19%)
Female	17 (81%)

**Figure 3:**
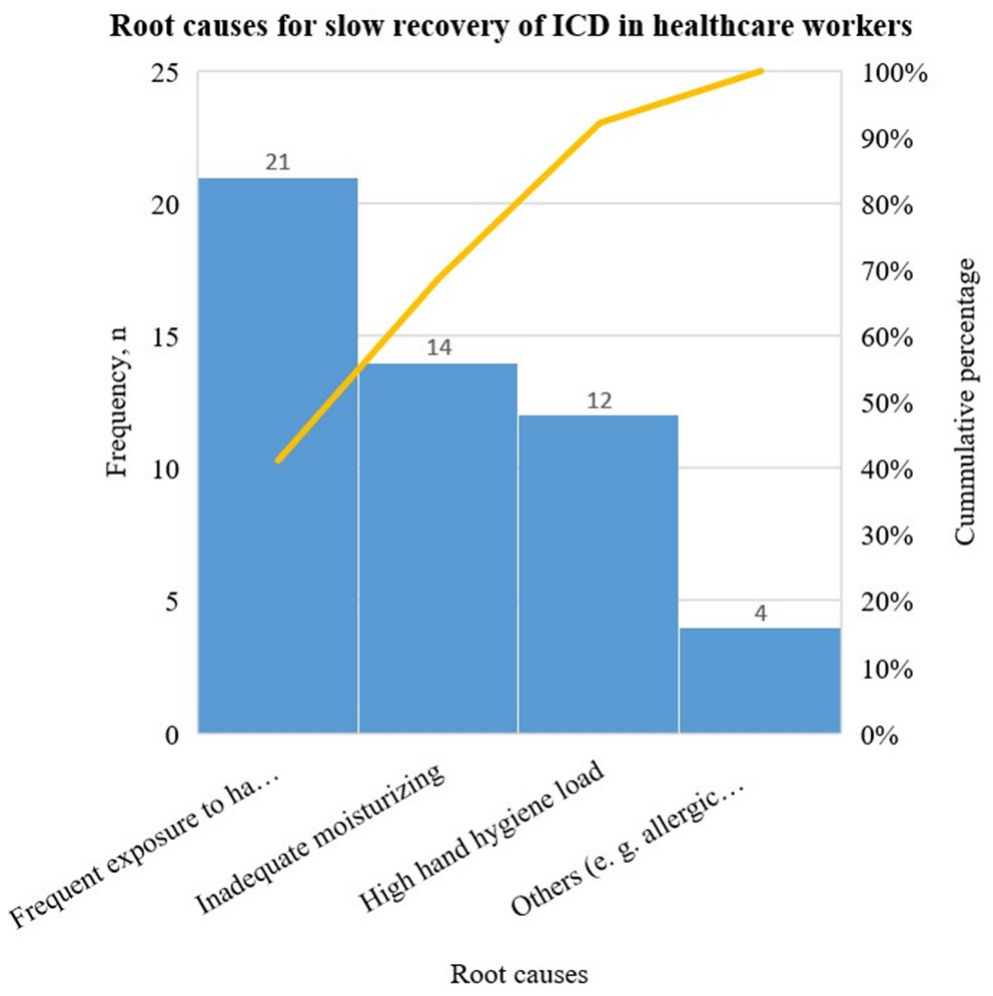
Pareto chart of the different root causes. Root causes: frequent exposure to hand hygiene products, inadequate moisturising, high hand hygiene load and others (e.g., allergic contact dermatitis). High hygiene load denotes daily hand hygiene activities of fifty times and above.

Frequent use of hand hygiene products was identified in all the participants, with 12 of them reported to have a high daily hand hygiene count of 50 times or more approximately. More than half were also found to moisturise their hands infrequently. Other factors that might contribute to the prolonged recovery time of ICD were found in only 4 participants (Figure 3).

During the first visit, 6 participants were found to have mild dermatitis and were not required to have temporary job modification. All participants were given medical letters detailing the intervention recommendations to pass to their superiors. At the 2-month post-intervention review, all participants were found to have a clinical improvement in their hand condition. Based on the visual analogue score, the estimated mean reduction of signs and symptoms was 80% in comparison to their baseline hand condition before intervention. The participants reported that their workplace supervisors were accommodating to the interventions by supplying the participants with the proposed hand hygiene products and making adjustments to reduce their clinical duties temporarily.

All participants had an improvement rate of 70–90% from their baseline signs and symptoms, with a mean improvement of 80%. Out of the 6 participants without job modification intervention, one participant had 70% clinical improvement, 3 participants had 80% improvement and 2 participants had 90% improvement. Out of the 15 participants with job modification intervention, 6 participants had 70% clinical improvement, 7 participants had 80% improvement and 2 participants had 90% improvement.

Clinically significant improvement in the hand condition of all the participants was noted during the 2-month post-intervention review with most of the participants returning to their full clinical duties after the intervention period.

Although their hand condition improved, none of the participants recovered completely after 2 months. Subsequently, all participants were given follow-up reviews at different intervals and were managed individually based on their clinical condition.

## Discussion

The use of gentler hand hygiene products at the workplace and temporary reduction in hand hygiene activities may be useful to aid the recovery of hand dermatitis in healthcare workers.

Based on the hierarchy of controls from the National Institute for Occupational Safety and Health (NIOSH), substitution of an irritant chemical with a less irritant alternative is the most effective control measure for managing a hazard at the workplace, after elimination (22). Elimination of hand hygiene activities is impossible for healthcare workers performing clinical duties due to the nature of their job.

Hand disinfection with an ABHR is the most common modality of infection control (24). However, while ABHRs are effective in preventing pathogenic transmission, the alcohol content in such formulations can be irritating and impair skin tolerability, which can result in reduced compliance to hand hygiene requirements (17, 24). A study found significant dryness and itching scores for workers using mixed gel which contains ethanol and isopropanol as compared to ethanol-only gel (25). Different ABHRs can have varying impacts on the skin depending on their composition. Emollients in ABHRs can also improve the skin condition and should be a factor when selecting ABHR for use (5, 25, 26).

Although ABHRs are generally better tolerated than hand washing with water and soap, considerations of their composition must be taken into account when deciding their tolerability (25). Hand washing remains an integral part of hand hygiene and is still recommended when hands are visibly soiled (17). Mild cleansers should be made available for healthcare workers at the workplace for hand washing purposes.

Temporary reduction of clinical workload with hand hygiene activities, while not curative, may aid in the recovery of irritant dermatitis by reducing exposure to the irritants and allowing a period of rest. It is considered a type of administrative control and may be less effective than the substitution of irritant products because the worker will still be continuously exposed to the irritant, albeit at a reduced rate. While implementation of temporary reduction in workload is feasible for a small number of affected healthcare workers, it is less sustainable in the long run, since the additional workload might be transferred onto other colleagues in the same unit. The additional hand hygiene load might put other colleagues at increased risk of developing hand dermatitis.

The use of protective items such as barrier cream and moisturiser can be considered the least effective method based on the hierarchy of control as it does not remove or reduce the hazard itself and can be affected by human behaviour. For example, the lack of accessibility to moisturisers or the additional time required to moisturise can be potential barriers.

While substitution with a less irritant ABHR and reduction in workload are beneficial for the recovery of ICD of the hands, complete avoidance of allergens is the treatment for ACD. Healthcare workers with ACD will need to be removed from further exposure to the offending allergen. Patch testing is often used to identify the offending allergen for individuals with ACD (27). However, patch testing is time-consuming and referral for patch testing will require an appointment with a dermatologist. At the height of the COVID-19 pandemic, resources were diverted to manage COVID-19 infections, and all non-urgent appointments were delayed. Referrals for patch testing were delayed beyond the 2 months' timeline set in the QI project. Since ICD accounts for a majority of hand dermatitis, workplace interventions targeting ICD and substitution of latex gloves; a common allergen, with other alternatives may be useful in improving hand dermatitis among healthcare workers while awaiting patch testing.

The strength of this study includes the assessment of the effects of workplace interventions based on principles of substitution and administrative controls to improve the recovery rate of occupational ICD. While previous studies done on healthcare workers focused on educational programmes and the use of moisturisers, they did not evaluate the effects of workplace interventions (28). A systemic approach including work-based changes is vital to prevent occupational hand dermatitis amongst healthcare workers while protecting the safety of patients.

Substituting highly irritant hand hygiene products with gentler alternatives may reduce the intensity of irritant exposure during hand hygiene activities. Furthermore, it can be implemented at a department level without incurring high expenditures.

There were several limitations in the study. The study lacks objective scoring of the hand dermatitis condition, such as the hand eczema severity index (HECSI) (29). Although a scoring index might be more useful in measuring objective changes, the process itself may be laborious and require input from a dermatologist. Photographic documentation for outcome assessment was performed to reduce the biases and the assessment was performed by the same OM physician to reduce inconsistency. The study also had a small sample size. A larger sample size would be beneficial in future studies to evaluate the effectiveness of different workplace interventions.

The workplace interventions were implemented through the participants' superiors *via* a medical letter. Although the superiors were generally supportive, the rate of implementation on the ground may vary: substitution with alternative ABHR or hand wash products might be affected by the supplies at the ward level, while temporary work adjustment will require time to implement due to manpower arrangement. The recovery from ICD might also be affected by factors outside of work, such as wet work activities from household chores.

Prevention and enhanced recovery from occupational dermatitis require disease awareness and early management. As part of primary prevention, appropriate control measures at the workplace can be implemented to reduce occurrences of hand dermatitis among healthcare workers at high risk of developing occupational dermatitis. Surveillance for early detection of the disease and individualised occupational management for affected healthcare workers can be performed to improve rate of recovery. To ensure a systemic and permanent workplace changes, support and collaboration with various stakeholders such as the hospital's management and the Safety and Health department on suitable workplace interventions will be necessary.

## Conclusion

Chronic occupational contact dermatitis can result in impaired quality of life and loss of work productivity. With increased incidences of ICD among healthcare workers, effective preventive measures should be implemented at the workplace.

Workplace interventions such as substituting highly irritant hand hygiene products with gentler alternatives and temporary reduction in clinical duties may be useful in improving the recovery rate of ICD among healthcare workers. Specific high-risk areas with high hand hygiene workload or high incidences of ICD may opt to implement systemic workplace changes to improve recovery and prevent new occurrences of ICD.

Further studies on the clinical effectiveness, sustainability and cost-benefits of different workplace interventions at a larger scale can be considered in the future. Effective systemic workplace changes can have significant positive impact on the worker and the workplace. Engagement and support from relevant stakeholders will be essential for sustained and effective change.

## Data Availability Statement

The datasets presented in this article are not readily available because they contain clinical details of the cases reviewed. Requests to access the datasets should be directed to AL, alicia.loi@mohh.com.sg.

## Ethics Statement

Ethical review and approval was not required for the study on human participants in accordance with the local legislation and institutional requirements. The patients/participants provided their written informed consent to participate in this study.

## Author Contributions

AL is the primary author responsible for the original draught and subsequent revision of the manuscript. She was involved in the design, implementation, and analysis of the study. ZA was also involved in the design and implementation of the study. She assisted in the analysis of the results and provided inputs during the revision of the manuscript. YF provided guidance and was involved in the design, implementation, and interpretation of the result. She reviewed the manuscript and provided direction on the drafting and revision of the manuscript. All authors have read and agreed to the final version of the manuscript.

## Conflict of Interest

The authors declare that the research was conducted in the absence of any commercial or financial relationships that could be construed as a potential conflict of interest.

## Publisher's Note

All claims expressed in this article are solely those of the authors and do not necessarily represent those of their affiliated organizations, or those of the publisher, the editors and the reviewers. Any product that may be evaluated in this article, or claim that may be made by its manufacturer, is not guaranteed or endorsed by the publisher.
